# Natural Time Analysis of Global Navigation Satellite System Surface Deformation: The Case of the 2016 Kumamoto Earthquakes

**DOI:** 10.3390/e22060674

**Published:** 2020-06-17

**Authors:** Shih-Sian Yang, Stelios M. Potirakis, Sudipta Sasmal, Masashi Hayakawa

**Affiliations:** 1 Institute of Space Science, National Central University, 300 Jhongda Rd., Jhongli District, Taoyuan 32001, Taiwan; 2 Department of Electrical and Electronics Engineering, University of West Attica, 250 Thivon and P. Ralli, Aigaleo, GR-12244 Athens, Greece; spoti@uniwa.gr; 3 Indian Centre for Space Physics, 43 Chalantika, Garia St. Road, Kolkata 700084, India; meet2ss25@gmail.com; 4 Hayakawa Institute of Seismo Electromagnetics, Co. Ltd., University of Electro-Communications (UEC) Alliance Center, 1-1-1 Kojima-cho, Chofu, Tokyo 182-0026, Japan; hayakawa@hi-seismo-em.jp; 5 Advanced Wireless & Communications Research Center, UEC, 1-5-1 Chofugaoka, Chofu, Tokyo 182-8585, Japan

**Keywords:** natural time analysis, time series analysis, criticality, crustal deformation, global navigation satellite system (GNSS), the 2016 Kumamoto earthquake, atmospheric gravity waves

## Abstract

In order to have further evidence of the atmospheric oscillation channel of the lithosphere-atmosphere-ionosphere coupling (LAIC), we have studied criticality in global navigation satellite system (GNSS) surface deformation as a possible agent for exciting atmospheric gravity waves (AGWs) in the atmosphere and GNSS fluctuations in the frequency range of AGWs with the use of the natural time (NT) method. The target earthquake (EQ) is the 2016 Kumamoto EQ with its main shock on 15 April 2016 (M = 7.3, universal time). As the result of the application of the NT method to GNSS data, we found that for the one-day sampled GNSS deformation data and its fluctuations in two AGW bands of 20–100 and 100–300 min, we could detect a criticality in the period of 1–14 April, which was one day to two weeks before the EQ. These dates of criticalities are likely to overlap with the time periods of previous results on clear AGW activity in the stratosphere and on the lower ionospheric perturbation. Hence, we suggest that the surface deformation could be a possible candidate for exciting those AGWs in the stratosphere, leading to the lower ionospheric perturbation, which lends further support to the AGW hypothesis of the LAIC process.

## 1. Introduction

To successfully predict the occurrence of a major earthquake (EQ) is an issue of mutual concern for human beings, since an EQ is one of the disastrous hazards that we have not been able to prevent for a long time. The searching and understanding of pre-seismic effects are required to make a successful EQ prediction. It has recently been agreed that precursory phenomena exist around the ground surface and in the lithosphere deservedly. However, several kinds of EQ precursors have been found far above the ground surface (e.g., [1,2]). The most important precursor is the perturbations in the ionosphere several days to 1–2 weeks before a forthcoming EQ. The ionospheric perturbations mainly appear in two ways. One way is the variations in the F-region electron density and is further seen in the total electron content (TEC) (e.g., [3,4,5,6]). The other way is the fluctuations in the bottom ionosphere observed using very low-frequency (VLF) and low-frequency (LF) radio wave techniques. More specifically, the VLF/LF propagation is found to be anomalous before many major EQs, within the following features [7,8,9,10]: (1) a decrease in nighttime amplitude, (2) an increase in nighttime fluctuation, (3) shifts in terminator (sunrise and sunset minima) times, and (4) a Doppler shift in VLF signals. These features, especially the Doppler effect, can be explained by the existence of pre-seismic atmospheric gravity waves (AGWs) and even acoustic waves (AWs) [11,12,13,14].

This AGW channel (also referred to as the atmospheric oscillation channel) is not the only possible agent that can explain the seismo-ionospheric precursors before a major EQ. There is also the chemical channel based on radon emanation [15,16] and the electrostatic channel that relies on the positive hole theory [17,18]. However, each of these hypotheses promises to be a good explanation to clarify how the pre-seismic effect(s) in the lithosphere can further propagate upward into the atmosphere and ionosphere, which is also called the lithosphere-atmosphere-ionosphere coupling (LAIC). Nevertheless, scientists have not yet concluded the final answer to the LAIC process, since the study of the LAIC is relatively recent, i.e., about two decades since the late 1990s.

Nevertheless, there have been numerous reports accumulated on the VLF/LF propagation anomalies before major EQs (see a review paper [19] and references therein, also refer to recent papers [20,21,22]) which support the AGW hypothesis of the LAIC process, and we consider that the precursory AGW acts as a responsible agent in the LAIC process.

The 2016 Kumamoto EQ, of interest in this paper, has been the most intense seismic event in Japan after the remarkable 2011 Tohoku EQ. During the EQ preparation period, the Japanese VLF/LF network, which is composed of eight receiving stations all around Japan, operated routinely, and the amplitude data enabled us to study the subionospheric features before the EQs in our previous paper [23]. We found that the lower ionosphere above the EQ epicenter was perturbed beginning about two weeks before the EQs. Further theoretical computations based on the wave-hop theory revealed that the effective reflection height of the VLF waves descended ~10 km during the most perturbed period of 3–5 days before the EQs. At the same time, the horizontal expansion of the perturbed region was about 1000 km centered on the EQ epicenter. According to the observational and theoretical results, we presumed that the lower ionosphere was disturbed by a large spatial scale agent, which was likely to be an AGW.

After [23], we further investigated the existence of precursory AGW activity in the neutral atmosphere [24]. The AGW potential energy (E_P_) was evaluated using the temperature profiles retrieved from the global ERA5 reanalysis dataset. The E_P_ in the stratosphere showed an obvious increase, i.e., the AGW activity had significantly enhanced around the EQ epicenter during the week before the EQs. Similar to the subionospheric perturbations, the active region of stratospheric AGW expanded and was most developed about 3–5 days before the EQs. The tempo-spatial coincidence of subionospheric perturbations with stratospheric AGW activity lends good support to our AGW presumption, but we still have to find certain evidence in the lithosphere in order to show that the anomalies in the ionosphere and middle atmosphere are pre-seismic effects, although it has been manifested that both anomalies occurred just above the EQ epicenter.

Then, our first attempt on the lithospheric features was the geomagnetic observation at Kanoya [25], about 150 km away from the EQ epicenter. The magnetic field variations of both horizontal and vertical magnetic components were surveyed in the ultra-low-frequency (ULF) band of 10–20 mHz (0.01–0.02 Hz), which has been considered to be the direct effect from the lithosphere [1,26]. However, we did not find any definite facts to show the presence of this lithospheric effect. Nevertheless, another analysis of the ULF/ELF (f = 0.1–24 Hz) impulsive emissions observed at the other three stations >500 km apart from the EQ epicenter, showed the presence of an indirect pre-seismic effect through the atmosphere.

To further explore any obscure lithospheric phenomenon hidden in the ULF data, we tried to employ the method of natural time (NT) analysis [27] to identify any approach to criticality [28]. The NT analysis was proposed to identify the hidden dynamical features in complex systems, such as the precursory seismo-electromagnetic signals [27]. This NT method was applied to the Kanoya magnetic data in our recent studies [29,30]. All the characteristic quantities in the ULF field and depression components were found to reach a criticality before the EQs, although the corresponding time periods were different between those quantities, ranging from two to four weeks before the EQs. Meanwhile, the same criticality analysis was performed on the subionospheric data as used in [23]. The results in [31] showed that the lower ionosphere underwent critical characteristics during the two weeks before the EQs, which was temporally consistent with the propagation anomalies analyzed using the conventional methods and theoretical computations in [23].

Here, we temporarily turn to the literature on another kind of lithospheric precursor, the crustal movements and resonance. The authors of [32] used global navigation satellite system (GNSS) position data to study the medium-term (on the time scale of months), short-term (days), and imminent-term (hours) crustal deformation, i.e., the variations of the geographic coordinates of latitude, longitude, and height, on both the pre-seismic and co-seismic variations of the 2011 Tohoku EQ. They found a discontinuity in the longitudinally crustal movement that occurred from eight days before the EQ, which was a short-term precursor in the lithosphere. Furthermore, a temporal overlap of precursory phenomena in the lower ionosphere [33] and stratosphere [34], before the same 2011 Tohoku EQ, was reported. Recently, the authors of [35] used GNSS position data, as well as seismometer data, and they found ground vibrations (crustal resonance) for a period of 3.5–5.5 h were enhanced 0–10 days before several major EQs.

On the basis of the successful use of the crustal deformation data in the past literature [32,35], it seemed to be a good idea for us to examine the crustal data to study the lithospheric effect before the 2016 Kumamoto EQs, since we did not get a definite conclusion on the lithospheric effect using the conventional ULF analysis [25]. In addition, the use of the NT method can further lend us to explore any hidden feature in the crustal data. Therefore, in the present study, we apply the NT analysis to the time series of deformation data, including the longitudinal, latitudinal, and vertical components, for the period from 13 March 2016 to 15 April 2016, which was about one month before the 2016 Kumamoto EQs. This is challenging and further strengthened by the fact that the observations of seismic electric signals (SES) activities [36] revealed that before the occurrence of major EQs there existed a crucial time scale of around a few months when long range correlations were developed or, at least, they were seriously affected. In other words, the SES activities observations [36] dictated that a few months before a main shock the critical stress was attained, which could reflect that changes in the correlation properties of other associated physical quantities, such as crustal deformation orientation, could become detectable at that time scale. The aim of this paper is to detect the occurrence of criticality in the lithosphere before the 2016 Kumamoto EQs, which could possibly be connected with our previous findings in the lower ionosphere [23] and stratosphere [24], as further support for the AGW mechanism of the LAIC process.

**Figure 1:**
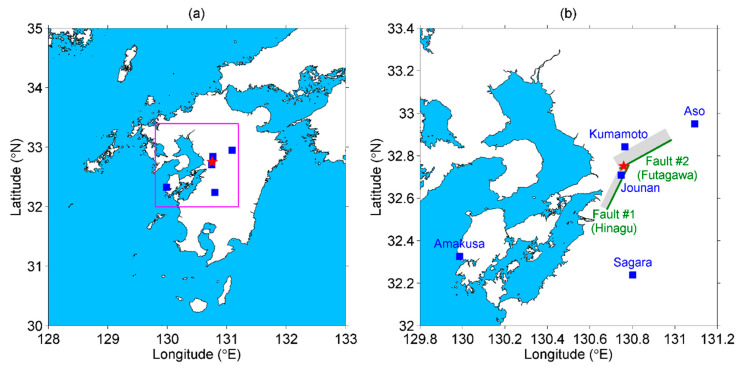
(**a**) The locations of the earthquake (EQ) epicenter (red star) and the five global navigation satellite system (GNSS) observing stations (blue squares) analyzed in this paper. The oceanic area is filled with blue color in the plot to clearly recognize the land and sea; (**b**) The enlargement of the squared area in (a), and the name of each GNSS stations are given. The green lines indicate the fault traces near the ground surface, and the gray shadows are the projection of ruptured fault planes under the ground. The EQ information and the fault traces were taken from [37].

Before ending this section, we provide some additional information on the targeted event in this study. A series of major EQs occurred in Kumamoto, Kyushu, Japan in April 2016. The first was a magnitude (M) 6.5 EQ that happened at 12:26 UT (21:26 local time) on 14 April, followed by several quakes and an M6.4 EQ at 15:03 UT on the same day. While the seismic activity seemed to decay after the two M6-class EQs, an M7.3 EQ occurred suddenly at 16:25 on 15 April. The M7.3 EQ was considered to be the main shock, and the two M6-class EQs were foreshocks of this seismic series. The geographic coordinates of the main shock are located at the geographic coordinates of 32.75° N, 130.76° E with 12 km depth, shown as a red star plotted in Figure 1a. Figure 1b is the enlargement of Figure 1a which only shows the crucial area of our interest. It also plots the two faults that were ruptured during the main shock. Faults #1 and #2 were ruptured along the pre-existing Hinagu fault and the Futagawa fault during the main shock, respectively [37]. This Futagawa-Hinagu fault system is considered to be part of the Median Tectonic Line (MTL). The MTL is the longest active and onshore tectonic structure in Japan going from Kagoshima Prefecture in southwest Japan, passing through Kumamoto Prefecture to Ibaraki Prefecture in east Japan [38,39].

## 2. Data

The present study is the first attempt to apply the NT method to a GNSS crustal deformation. In this section, we explain the data and our experimental designs in detail.

### 2.1. The Global Navigation Satellite System (GNSS) Observations

Plates drift due to several mechanisms between the Earth’s lithosphere and asthenosphere. Stresses accumulate around the boundaries of plates and further cause EQs via stress releasing [40]. Although the stress accumulation of plates under the ground is invisible, it can be observed by monitoring the deformation and movement of the crust. In the present study, the crustal deformation is defined and measured as the variations in the three geographic coordinates of latitude, longitude, and height that are obtained from the GNSS data. An example of the conventional deformation analysis on the pre-seismic and co-seismic effects can be found in [32].

The Japanese GNSS Earth Observation Network System (GEONET) is one of the geodetic survey projects operated by the Geodetic Observation Center, Geospatial Information Authority of Japan (GSI). The GEONET is composed of more than 1300 continuously operating reference stations (CORS) that cover the Japanese archipelago. The object of this system is to provide geodetic surveys and also crustal deformation information for EQ and volcanic studies. The GEONET data are available through the GSI website (http://datahouse1.gsi.go.jp/terras/terras_english.html).

There are 24 GEONET stations over Kumamoto Prefecture. However, these stations are very close (< 20 km) to each other. Physical parameters such as strain rate, gravity, and thermal signature are usually continuously distributed in space in the crust, except for a region with different tectonic structures, and these physical parameters can evidently change around structural boundaries (e.g., [41,42]). It is expected that the neighboring stations with the same tectonic characteristics have similar crustal deformation and criticality results. Therefore, in the present study, we only analyzed five representative stations with different tectonic conditions using the NT method.

These five stations (as indicated in Figure 1) with a short description of the tectonic properties, respectively, are listed from the closest to the farthest station to the EQ epicenter as follows:

(i) Jounan station (GEONET station ID: 021071; geographic coordinates 32.71° N, 130.75° E; and distance to the EQ epicenter 5 km) was the closest station to the EQ epicenter among all stations in Kumamoto Prefecture.
(ii) Kumamoto station (950465; 32.84° N, 130.76° E; 10 km) was the second closest station. It was located at the hanging (northwest) side of Fault #2, whereas the Jounan station was located at the foot (south to southeast) side of Fault #2 but the hanging (northwest) side of Fault #1.
(iii) Aso station (960703; 32.95° N, 131.09° E; 38 km) was located at the mountainside of the Aso volcano (Mount Aso) over the MTL. It was also located around the northeast end of Fault #2.
(iv) Sagara station (950469; 32.24° N, 130.80° E; 57 km) was located at the Hitoyoshi Basin. The Hitoyoshi Basin is a volcanic rift around the Butsuzo Tectonic Line (BTL), a fault system that was active during the Tertiary Period.
(v) Amakusa station (950467; 32.33° N, 129.99° E; 86 km) was located at the southwestern extension of the Futagawa-Hinagu fault system near the MTL. This station was situated in the Amakusa archipelago where it was separated from the rest of Kumamoto Prefecture by the Yatsushiro Sea.

### 2.2. Positioning of the GNSS Data

The GEONET system receives several GNSS and RNSS (regional navigation satellite system) missions, including the famous American Global Positioning System (GPS) as well as the European Galileo system, the Russian Global Navigation Satellite System (GLONASS), and the Japanese Quasi-Zenith Satellite System (QZSS). The raw data are recorded with a 30 s interval in receiver independent exchange format (RINEX). The RINEX files contain navigation data (commonly known as N files) and observation data (O files). The GNSS satellites broadcast coded time signals and orbital data using L-band (1–2 GHz) electromagnetic waves. Since the waves travel at the speed of light, a user can obtain the time-of-flight information by way of receiving these coded signals transmitted from different satellites. That information is stored in the observation data, but no direct position information is included in the RINEX files. Therefore, a preprocessing procedure is needed before we apply the NT method to the crustal information.

Several kinds of algorithms can derive the position of the user (receiver) within various accuracies ranging from meter level to centimeter level. However, the detail of GNSS techniques is beyond the scope of this paper. Readers are referred to some professional books on GNSS techniques (e.g., [43,44]), and we do not go into the details but provide a brief introduction to the positioning method we used in the present study.

Our goal of this paper is to identify any precursory evidence hidden in the lithosphere. Hence, the most precise information on crustal deformation is needed. For the centimeter-level purpose, one can use the conventional real-time kinematic (RTK) positioning method to acquire the precise position of the surveying receiver. However, the RTK method requires a base station (stationary receiver with known position) to obtain the precise position of the surveying receiver (either stationary or moving) with centimeter-level accuracy. In such a situation, the location of the surveying station relies on the base station. Once we find a precursory variation from the crustal information, we are not able to determine whether the precursor appears at the surveying station or the base station. The RTK method does not seem feasible in the present study. Therefore, we need another positioning method that requires only the information from the surveying station, and we can further conclude that the observed precursor has appeared at a specific station.

Therefore, here, we used the precise point positioning (PPP) technique instead of the conventional RTK method. The PPP method provides an easy way to acquire the precise position using a sole GNSS receiver, and no base station is needed [44]. In the present study, we used the online PPP tool provided by the Natural Resources Canada (NRC; https://webapp.geod.nrcan.gc.ca/geod/tools-outils/ppp.php). NAD83(CSRS) v7 was set as the reference frame during the online PPP calculation. NAD83(CSRS) is a geodetic reference system released by the Canadian Geodetic Survey (CGS). The 7th version of NAD83(CSRS) is improved from the latest International Terrestrial Reference Frame (ITRF), ITRF2014 [45]. Epoch was set at 2016.0 as the GNSS data in this study.

**Figure 2:**
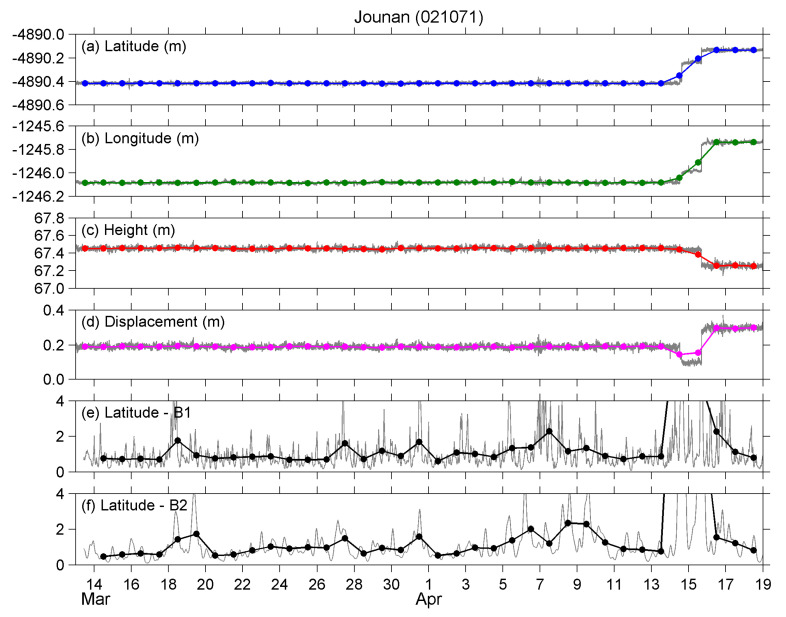
The time series of (**a**) latitude; (**b**) longitude; (**c**) height; (**d**) displacement; (**e**) B1 band atmospheric gravity wave (AGW) components of latitude; and (**f**) B2 band AGW components of latitude, respectively, at the Jounan station. The gray curve in each panel plots the 30 s data, while the bold colored curve in the top four panels and the bold black curve in the bottom two panels are the 1-day (daily mean) values of the time series.

The original RINEX observation data at the five select stations from 13 March 2016 to 15 April 2016 were downloaded from the GSI website. Then, the data were inputted to the NRC online PPP tool, and thus we obtained the positioning results (three geographic coordinates) derived by the PPP method, which were latitude (Lat), longitude (Lon), and height (Hei). The gray curves in Figure 2a–c show how the PPP results varied with time at the Jounan station. The results during the period 16–18 April are also plotted for reference, although our analyses were limited to the period from 13 March to 15 April. The latitude and longitude were converted to a local tangent plane coordinate with the unit of meter, as shown in the figure. The origin of the two horizontal coordinates was defined as the EQ epicenter, and positive values of latitude and longitude were indicated northward and eastward, respectively (i.e., the ENU/NEU coordinate system).

We also defined the displacement (Displ). The mean values of the three coordinates during the analyzed period (〈Lat〉,〈Lon〉, and 〈Hei〉) were calculated at each station, and then the mean values were subtracted from the instant values to get the deviations at the three coordinate components, i.e., $Lat^{\prime }=Lat-\langle Lat\rangle$, $Lon^{\prime }=Lon-\langle Lon\rangle$, and $Hei^{\prime }=Hei-\langle Hei\rangle$. The displacement was defined as the magnitude (vector sum) of the three deviation terms that $Displ=\sqrt{Lat^{\prime }{}^{2}+Lon^{\prime }{}^{2}+Hei^{\prime }{}^{2}}$. As an example, the displacement at the Jounan station is shown in Figure 2d by a gray curve. We notice that the variations in all the deformation parameters (Figure 2a–d) are quite small and difficult to recognize in the figure until the co-seismic crustal movements happened on the 14 (two violent foreshocks) and 15 (the main shock) of April.

### 2.3. The Atmospheric Gravity Wave (AGW) Fluctuation Components of the GNSS Surface Deformation Data

Since we are searching for evidence which can support the AGW hypothesis of the LAIC process, in the present study, we tried to retrieve the AGW components from the surface deformation data, including the three coordinate components and displacement, as described in Section 2.2, although the deformation is lithospheric information and not atmospheric information.

Our previous study [24] showed the existence of stratospheric AGWs before the Kumamoto EQ. However, the wave period of the precursory AGW could not be determined because the temporal resolution of the dataset, ERA5 atmospheric reanalysis, was not sufficient for such determination of the AGW wave period. Except for the Kumamoto EQ, regarding two intense EQs (M6.8 and M7.0) over eastern Japan, the AGW activity in the lower ionosphere with wave periods between 10 and 100 min was increased several days before the EQs [7]. The results lend us a good course on the searching of AGW activity hidden in the deformation data.

We used wavelet transform to obtain the scalogram representation of the 30 s data of Lat, Lon, Hei, and Displ. Scalogram, as a time-scale analysis method, provides both time- and scale-domain information, where the scale is analogous to the period (inverse of frequency) of Fourier analysis. The wavelet spectra revealed two facts that further affect our methodology in this section. First, the wavelet power varied with scale, i.e., low-frequency (long-scale) components had larger power than that of high-frequency (short-scale) components. Second, the power of high-frequency components, especially the ones at <10 min periods, varied with time violently. For the first point, the power at each scale (frequency) was normalized using the mean value (1 = mean value), so the normalized power was equivalent at different scales (frequencies). For the second point, and also considering the conclusions by [7], we defined the two frequency bands hereafter referred to as “B1” corresponding to 20–100 min scales and “B2” corresponding to 100–300 min scales. The B1 band had the same upper limit as the finding in [7], but the lower limit was changed to 20 min. It means we discarded the 10–20 min components, which could contain some high-frequency noise in the data. In addition, we added the B2 band to see if there was any information hidden at longer scales of 100 to 300 min. Similar to the deformation graphs in Figure 2a–d, the gray curves in Figure 2e,f plot the normalized power of the B1 and B2 bands of latitude at Jounan, respectively.

### 2.4. Resampling

Since this study is the first time that NT analysis has been applied to GNSS surface deformation and AGW time series, our first concern was to appropriately define the “events” necessary for the application of the specific analysis method (see details in Section 3). The positioning results have a good temporal resolution of 30 s, similar to the raw data. The NT analysis could be applied to any discrete time series with fixed (or not fixed) time spacing. However, we did not need to perform the NT analysis with the 30 s data, because the pre-seismic processes related to the LAIC usually had a much longer time scale of a day to a few weeks (e.g., [3,5,9]).

Nevertheless, we also expected that lithospheric criticality information, if any, could be more natural to be identified on coarse-grained data, since criticality has already been determined before the Kumamoto EQs using the NT method applied to the one-day resolution, i.e., daily-valued time series of other observable parameters related to LAIC, such as VLF subionospheric propagation quantities and specific ULF magnetic field quantities calculated from ground-based magnetometer recordings [23,25,29,31]. It is worthwhile using the same one-day data in the present study, and we can further compare the results of the crustal deformation with that of VLF subionospheric perturbations and ULF magnetic variations (Section 5) using the same data sampling rate.

In addition, our previous study regarding the stratosphere [24] reported abnormal AGW activity during 9–15 April before the Kumamoto EQ, which is direct evidence that precursory AGW exists in the middle atmosphere. The one-day sampling is sufficient to recognize the evolution of stratospheric AGW activity, as well as the crustal criticalities detected by the NT method, because, in the present study, we are trying to find the possible lithospheric agent of exciting precursory AGWs.

On the basis of the above two points, we averaged the 30 s values (the gray curves in Figure 2a–f) to get the one-day values of the geographic coordinates (the bold blue/green/red curve in Figure 2a–c), displacement (the bold magenta curve in Figure 2d), and AGW fluctuations (the bold black curves in Figure 2e,f). Then, the daily values, as inputs, were analyzed using the NT method to identify any criticalities at the five stations.

One remark has to be mentioned here. The VLF subionospheric perturbations are observationally more evident during nighttime than daytime [9]. In our previous papers regarding VLF propagation anomalies before the 2016 Kumamoto EQs [23,31], the one-day values were calculated using only the nighttime (mainly during 19:00–24:00 and 00:00–05:00 on the subsequent day in local time) data, but not the whole day data. Nevertheless, the results are quite valuable for this study and are discussed with the findings in the present study in Section 5.

## 3. Natural Time (NT) Analysis

The transformation of a time series of “events” from the conventional time domain to the NT domain is performed by ignoring the time interval between events and retaining only its normalized order of occurrence [27]. Accordingly, the NT of the k-th event is defined as $\chi_{k}=k/N$, where N is the total number of successive events. The “energy” $Q_{k}$ of each event is retained. Then, the transformed time series ($\chi_{k}$,$Q_{k}$) is studied. By defining $p_{k}=\frac{Q_{k}}{\sum_{n=1}^{N}Q_{n}}$, the energy of k-th event normalized by the total energy, a system is considered to approach a criticality when the parameter $\kappa_{1}=\overset{N}{\underset{k=1}{\sum }}p_{k}\chi_{k}^{2}-{\left(\overset{N}{\underset{k=1}{\sum }}p_{k}\chi_{k}\right)}^{2}$ converges to the value $\kappa_{1}=0.070$ and at the same time both the entropy in NT, $S_{nt}=\overset{N}{\underset{k=1}{\sum }}p_{k}\chi_{k}ln\chi_{k}-\left(\overset{N}{\underset{k=1}{\sum }}p_{k}\chi_{k}\right)ln\left(\overset{N}{\underset{k=1}{\sum }}p_{k}\chi_{k}\right)$ and the entropy under time reversal, $S_{nt-}$, satisfy the condition $S_{nt},S_{nt-}<S_{u}=\left(\frac{\ln2}{2}\right)-\frac{1}{4}\left(\approx 0.0966\right)$, where $S_{u}$ stands for the entropy of a “uniform” distribution in NT [29,46]. This is the set of criteria for criticality, according to the NT analysis, that is usually applied to time series (e.g., [29]), and has been applied to different raw (unprocessed) electromagnetic recordings possibly related to EQ such as the ultra-low-frequency (≤1 Hz) SES [36,47,48,49,50] and the fracto-electromagnetic MHz signals [51,52,53,54]. It has to be mentioned that the criterion of the $\kappa_{1}=0.070$ value has originally been derived for SES activity and, later, on the basis of the Ising model. Its validity has been confirmed on real SES time series, while it has also been verified to be valid for several self-organized criticality (SOC) models and real-time series of a variety of applications. In all these dynamical systems, it has been found that the value $\kappa_{1}=0.070$ can be considered to be quantifying the extent of the organization of the system at the onset of the critical stage [27].

Moreover, the NT analysis is also applied to daily-valued quantities, such as specific ULF magnetic field quantities calculated from ground-based magnetometer recordings [29,55,56,57], and VLF subionospheric propagation quantities [31], although in such cases there is usually a limited number of available data, as happens with the NT analysis of foreshock seismicity. However, in such cases, a criticality is checked differently following the paradigm of the NT analysis of foreshock seismicity [46,48,50,58], while in more complex systems, the identification of the approach to criticality requires the study of the evolution of the entropy change ΔS ($=S_{nt}-S_{nt-}$) under time reversal [59].

Specifically, concerning the application of NT analysis to seismicity, the temporal evolution of specific NT analysis parameters is studied by progressively including new events in the analysis and each time calculating $\kappa_{1}$, $S_{nt}$, $S_{nt-}$, and 〈D〉 based on the events already included. Note that 〈D〉 is the “average” distance $\langle D\rangle =\langle |\Pi \left(\varpi \right)-\Pi_{critical}\left(\varpi \right)|\rangle$ between the curves of normalized power spectra $\Pi \left(\varpi \right)={\left|\overset{N}{\underset{k=1}{\sum }}p_{k}exp\left(j\varpi \chi_{k}\right)\right|}^{2}$ (ϖ is the natural angular frequency, ϖ=2πφ, with φ standing for the frequency in NT, termed “natural frequency”) of the evolving seismicity and the theoretical estimation of Π(ϖ) for $\kappa_{1}=0.070$, $\Pi_{critical}\left(\varpi \right)\approx 1-\kappa_{1}\varpi^{2}$ [27]. Of course, in each step the time series ($\chi_{k}$,$Q_{k}$) is rescaled in the NT domain, since each time the $k^{th}$ event corresponds to a NT $\chi_{k}=\frac{k}{N}$, where N is the progressively increasing total number of the considered successive events; then, all the parameters involved in the NT analysis are calculated for this new time series; this process continues until the time of occurrence of the main EQ event. In the resultant time evolution of $\kappa_{1}$, $S_{nt}$, $S_{nt-}$, and 〈D〉, criticality is considered to be truly achieved when, at the same time [27,47] the following occurs: (i) $\kappa_{1}$ approaches $\kappa_{1}=0.070$ “by descending from above”, (ii) $S_{nt},S_{nt-}<S_{u}$, (iii) $\langle D\rangle <10^{-2}$, and (iv) since the underlying process is expected to be self-similar, the time of criticality does not significantly change by varying the “magnitude” threshold. Although the selection of thresholds is arbitrary in the case of ULF magnetic field and VLF subionospheric propagation quantities (usually more than 20 threshold values equispaced between zero and a maximum threshold value larger than the 50% of the maximum value of the examined quantity are considered), if criticality conditions are met in close dates for more than one of the considered threshold values, then, this is considered to be an indication of the validity of the performed analysis. It may be worth mentioning that these criticality conditions, including the approach to $\kappa_{1}=0.070$ “by descending from above”, were not derived theoretically but empirically from computing $\kappa_{1}$ values for well-known phenomena and actual data of seismicity in Greece.

## 4. NT Analysis of GNSS Deformation Data and AGW Data

We considered that the first derivative of the original data would better reveal the underlying dynamics, since it focused on temporal change. In order to define positive valued events, we also applied absolute value as the final stage of the original data preprocessing. We analyzed the absolute value of the first derivative of the one-day sampled time series of GNSS components and displacement, as well as the AGW components of these parameters.

As mentioned in Section 2, the analyzed period was from 13 March to 15 April, while the criticality criteria are the ones mentioned in Section 3 for the case of foreshock seismicity and other cases of a limited amount of available data.

**Figure 3:**
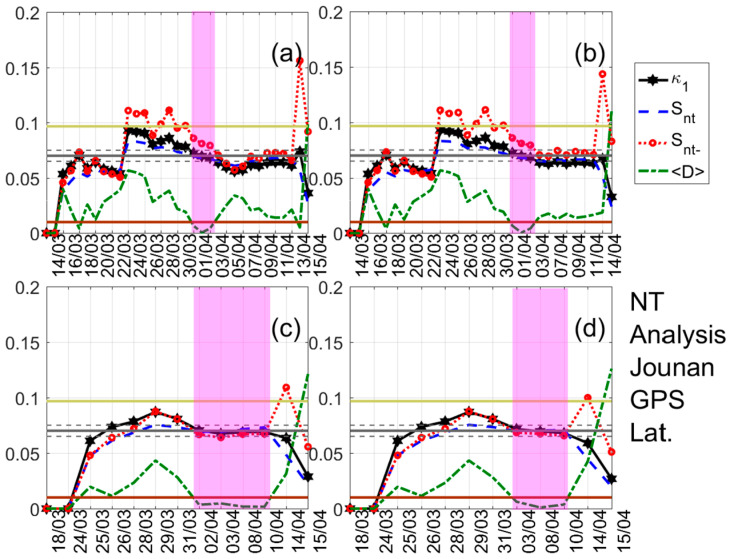
Natural time (NT) analysis of the absolute value of the first derivative of the one-day sampled version of the latitude GNSS component recorded at the Jounan station. Variations of the NT analysis parameters for four different thresholds. (**a**) 0; (**b**) 0.22; (**c**) 0.88; and (**d**) 0.99. The entropy limit of $S_{u}\left(\approx 0.0966\right)$, the $\kappa_{1}$ value 0.070, and a region of ±0.005 around it are shown by the horizontal solid light green, solid grey, and the grey dashed lines, respectively. The horizontal solid brown line denotes the 〈D〉 limit ($10^{-2}$). According to the NT analysis method, the shaded areas indicate the time range when criticality conditions are satisfied (cf. Section 3). Note that the events employed depend on the considered threshold. Moreover, the time (x-) axis is not linear in terms of the conventional date of occurrence of the events, since the employed events appear equally spaced relative to the x-axis as the NT representation demands, although they are not equally spaced in conventional time.

**Figure 4:**
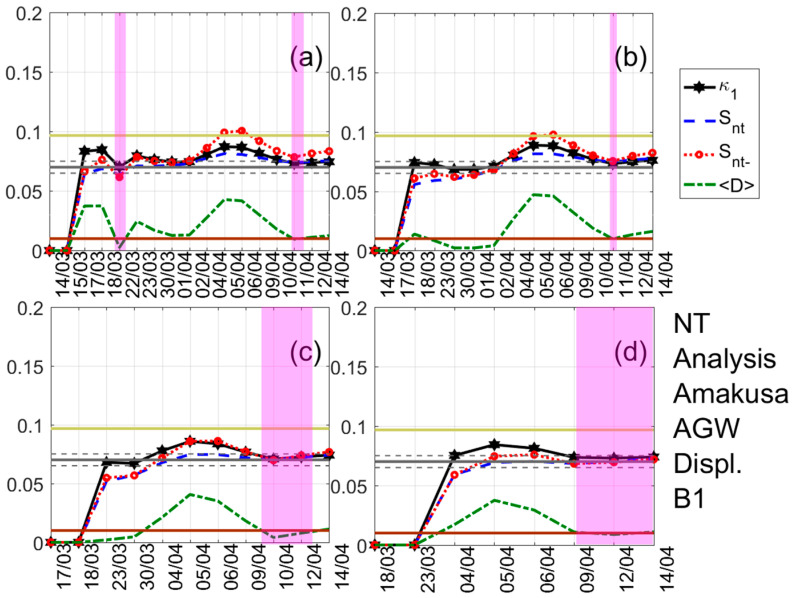
NT analysis of the absolute value of the first derivative of the one-day sampled displacement AGW time series of the Amakusa station for the B1 band. Variations of the NT analysis parameters for four different thresholds. (**a**) 0.175; (**b**) 0.225; (**c**) 0.325; and (**d**) 0.35. Figure format similar to Figure 3 format.

**Figure 5:**
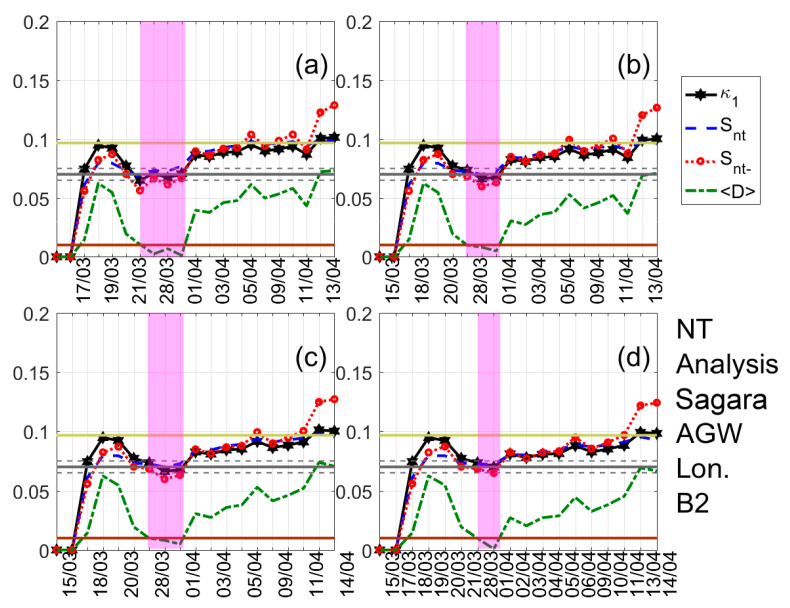
NT analysis of the absolute value of the first derivative of the one-day sampled longitude AGW time series of the Sagara station for the B2 band. Variations of the NT analysis parameters for four different thresholds. (**a**) 0.175; (**b**) 0.225; (**c**) 0.325; and (**d**) 0.35. Figure format similar to Figure 3 format.

A typical example of the results obtained after the application of NT analysis is shown in Figure 3. It shows the temporal evolution of the NT parameters $\kappa_{1}$, $S_{nt}$, $S_{nt-}$, and 〈D〉 for four threshold values of the latitude GNSS component from the Jounan station. Each time a value of the analyzed time series exceeds the corresponding threshold, a new event is included in the analysis, and all the studied NT parameters are re-calculated. Magenta patches indicate the time period during which the NT analysis criticality conditions are satisfied for each threshold value. One can observe that during these periods, $\kappa_{1}$ approaches the value $\kappa_{1}=0.070$ “by descending from above”, $S_{nt},S_{nt-}<S_{u}\left(\approx 0.0966\right)$, and $\langle D\rangle <10^{-2}$, simultaneously, satisfying the criteria (i)–(iii) (see the application of the NT analysis to seismicity in Section 3) for the approach to criticality. The four time periods are overlapping, intersecting on 3 April which indicates that the critical state is truly achieved on that day, since for this date the criterion (iv) (see the application of the NT analysis to seismicity in Section 3) is also satisfied. Figure 4 and Figure 5 show examples of the NT analysis results, for the cases of the displacement AGW time series of the Amakusa station for the B1 band and the longitude AGW time series of the Sagara station for the B2 band, respectively.

**Figure 6:**
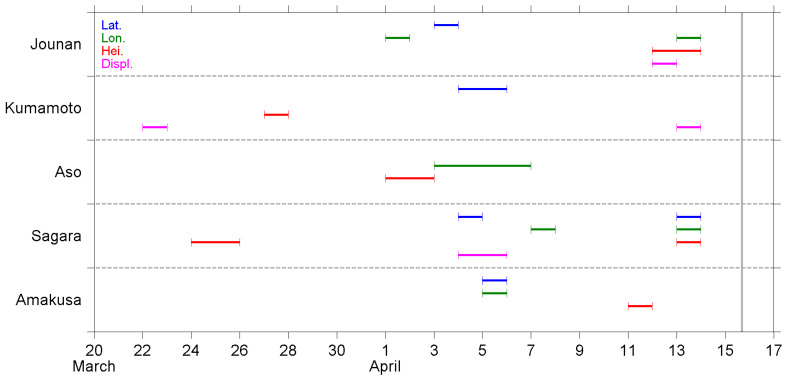
The time of approach to criticality for the GNSS deformation time series. The vertical line marks the time of the Kumamoto EQ (main shock). The horizontal dashed lines divide the figure into five partitions, and each partition contains the results at one station as marked on the left hand side. There are several segments with different colors in each partition. The blue, green, red, and magenta segments show the time of criticality on latitude, longitude, height, and displacement, respectively.

**Figure 7:**
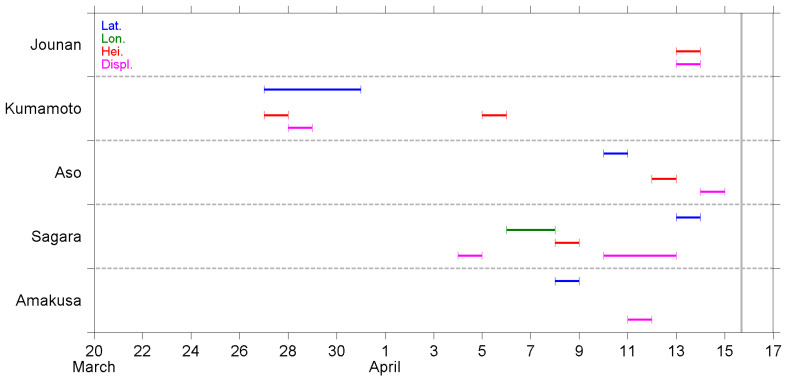
Same as in Figure 6, but for the results of the B1 band (20–100 min) fluctuations retrieved from the deformation data.

**Figure 8:**
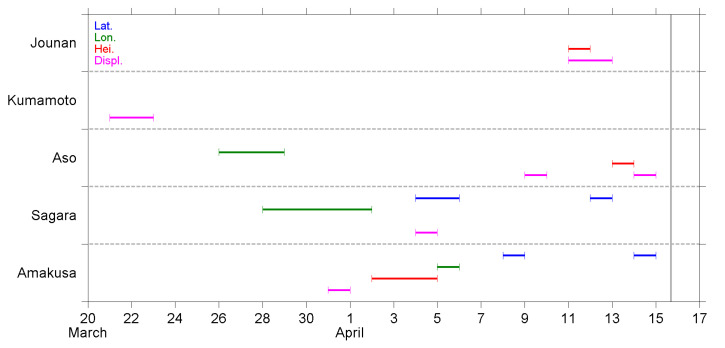
Same as in Figure 6, but for the results of the B2 band (100–200 min) fluctuations retrieved from the deformation data.

A summary of the NT analysis results is shown in Figure 6, Figure 7 and Figure 8. The results, as observed at different stations, are shown in the same figure, while the order of appearance in the figure is sorted by the distance between the station and EQ epicenter, Jounan station being the closest to the epicenter (see Section 2.1).

Figure 6 portrays the dates during which each GNSS deformation time series was found to approach a criticality. A dispersion of criticality dates is observed as an overall picture. However, the criticality dates can be mainly grouped into three stages. At the first stage in late March, the displacement at the Kumamoto station and height at the Sagara station, as well as at the Kumamoto station, approached critical states sequentially. Later on, numerous time series approached their criticality at the second stage in early April, and all the four deformation parameters were found to approach critical states, although their occurrence was observed at different stations. Among those parameters, latitude and longitude were the most prevalent parameters, both of them approached criticality at four stations. More specifically, at Jounan, the criticality of latitude occurred two days after that of longitude. However, the two parameters provide a different story at the Sagara station, where the latitude criticality led longitude criticality by three days. Moreover, the two parameters approached criticality on the same day at the Amakusa station. The results exhibit a great deal of variety. Nevertheless, at least one and up to three kinds of criticality were observed at each station during the second stage. Lastly, the third stage appeared just a few days before the EQ. Critical states were approached multiple times at the stations except for Aso on 11–13 April. Especially on 13 April, six time series presented criticality. We also noticed that the latitude and longitude at the Sagara station, as well as the displacement at the Kumamoto station, approached criticality twice at different stages, although in some cases, criticality was first approached for a lower threshold, and then approached again for a higher threshold.

Figure 7 presents the criticality dates for the time series of the B1 band (20–100 min) fluctuations. Criticality first appeared on the latitude, height, and displacement at the Kumamoto station on 27–28 March. Except for those, all the rest of the criticalities were observed after 4 April, i.e., within 10 days before the EQ occurred. There is no evident tendency for the occurrence of criticality on these 10 days. However, it seems 13 April was again an important date because there were three time series that approached criticality on this day. One more remark on the occurrence is that the criticality on longitude only approached once at the Sagara station, but the criticality on displacement was observed at every station, although the dates were different. Additionally, the height at the Kumamoto station and displacement at the Sagara station approached criticality twice.

Figure 8 shows the criticality dates, as shown in Figure 7, but for the B2 band (100–300 min) fluctuations. Similar to the B1 band, the criticality at the Kumamoto station occurred much earlier than other stations. Only the displacement, but not the other three parameters, at the Kumamoto station approached criticality. The criticality dates at the other four stations were highly dispersed and were observed over three weeks from late March to the day before the EQ occurred. Again, the criticality on displacement was approached at every station, although the occurrence dates were widely spread. The displacement at the Aso station, as well as the latitude at the Sagara and Amakusa stations, approached criticality twice.

## 5. Discussion

In the present study, we performed a criticality analysis using the NT method regarding the GNSS deformation data and two spectral components in the range of AGW. The daily time series at five GNSS stations were analyzed, and the approach to the critical state was recognized as the results shown in Figure 6, Figure 7 and Figure 8. Since this is the first time that the NT analysis has been applied to GNSS deformation data, we have some discussions and comments on the results and their meaning.

First of all, we need to discuss how realistic the findings are in the present study, i.e., are the results in Section 4 reasonable and tenable? Scientists have used GPS data to study crustal deformation for about 25 years. The techniques are well developed but mainly applied to study co-seismic deformation and long-term (>1 year) movement of plates (e.g., [60,61]). The long-term movement of plates is also a kind of pre-seismic crustal deformation. However, this kind of long-term variation is different from the precursory effects (from medium term to imminent term, on the time scale of months to hours) that we are concerned with. Nevertheless, the crustal deformation, as derived from GPS/GNSS data, is still a useful tool for EQ precursory studies, and it has been analyzed using conventional vector analysis, as well as time-frequency analysis, in previous studies. As introduced in Section 1, short-term precursors on the crustal deformation were found within about one week before the 2011 Tohoku EQ [32]. For the same EQ, the authors of [62] also used GPS data and found that the disturbance of compressive stress on the shallow crust appeared from 65 days to 47 days before the EQ. Their methodology was based on analyses using the Hilbert–Huang transform, one kind of time-frequency analysis that is popular for seismic signal analyses, and its theorem is much different from the NT analysis we used in the present study. Their analytical and statistical studies over central Taiwan [63,64] used horizontal azimuths converted from surface deformation at numerous GPS/GNSS stations, and found that the orientations of azimuths turned in a similar direction, specifically, in agreement with the direction of most compressive axes of the EQ-related loading stress, during the period of several days to weeks before the EQs. In summary, the previous studies [32,35,63,64] evidenced the precursory anomalies regarding crustal and surface deformation, during the time periods of about a few days to weeks before the forthcoming EQs. Similar to previous literature, we found that the deformation parameters (Figure 6) started to approach a critical state about one month before the 2016 Kumamoto EQ. Most criticalities were observed within the period two weeks before the EQ, in two stages; the first stage was in early April and another stage was just two to four days before the EQ. The investigation regarding AGW band perturbations in the lower atmosphere was still absent. However, some papers have reported the AGW perturbations in the stratosphere and lower ionosphere (e.g., [7,24,34]) as well as the ground vibrations for a period of 3.5–5.5 h (also in the range of some AGWs) [35] from a few to 10 days before the EQs. The results of the present study are in agreement with anomalous periods as found in previous papers, which means our results are realistic to show the short-term precursor before an EQ.

Next, we discuss the possible physical meaning of the results as shown in Figure 6, Figure 7 and Figure 8. We analyzed the four deformation components of latitude, longitude, height, and displacement; the former three components are geographic coordinates and the last component is a composite term of the former three. From the point of view regarding the excitation of AGWs, the essence of the wave is the oscillation of fluid (air parcel) caused by gravitational force and the restoring buoyancy force. The bouncing of fluid is mainly in, but not limited to, the vertical direction. On this basis, we give more attention to the vertical variation of the geographic coordinate, i.e., the height component, than the others in our analysis and study. The critical states of height deformation were observed at every station, although the dates were spread (Figure 6). However, not all the time series of B1 and B2 height fluctuations showed a criticality (Figure 7 and Figure 8). While we focused on the period about one week before the EQ (8–14 April), criticality was approached at the Jounan, Sagara, and Amakusa stations for the height deformation (Figure 6); at the Jounan, Aso, and Sagara stations for the B1 height component (Figure 7); and at the Jounan and Aso stations for the B2 height component (Figure 8). The criticality approach on crustal deformation means that the ground surface or the lithosphere is at a critical state before the EQ, ready to trigger some phenomena around the ground surface. Similarly, the B1 or B2 fluctuations showed a criticality, which means the crust would be ready to excite motions within the B1 or B2 period, such as observed by [35], and further atmospheric oscillations around the ground surface. Since the precursor AGW activity appeared in the stratosphere almost during the same period of one week before the EQ [24], the criticality results plausibly reveal the connection between ground motions (especially in the vertical direction) and the excitation of AGWs.

Although the results of the height time series seem to be enough to support our argument of surface deformation on AGW excitation, we investigated the approach to criticality of the other three deformation components and the results are discussed here. The criticalities of latitude and longitude were also observed at the Jounan station, and more frequently at the distant stations of Sagara, as well as the Amakusa station, no matter the time series of deformation or B1/B2 fluctuations. In addition, some of those critical states on latitude and longitude were approached earlier than that on height. Considering that the ruptured faults of the Kumamoto EQ were strike-slip faults (but the fault planes were oblique, as shown in Figure 1b), the crustal walls moved horizontally. It is reasonable that criticalities also appear on the two horizontal geographic coordinates of latitude and longitude. The displacement is a vector sum of the deviations of the three geographic coordinates, so the criticalities on displacement can either repeat the result of a certain coordinate or enhance the sensitivity of criticality detection.

Furthermore, we found that Jounan was the only station that the three height time series (deformation, B1, and B2 fluctuations), as well as the displacement time series, all approached criticality on the consecutive days of 11–13 April. However, numerous time series at the remaining four stations approached criticalities on 11–13 April. These days were the most important days for criticality before the occurrence of the Kumamoto EQ. These results could be some kind of short-term (2–5 days before the main shock, or 1–4 days before the two M6-class foreshocks) warning that a major EQ was incubating.

Both the Jounan and Kumamoto stations are pretty close to the EQ epicenter (within 10 km distance). However, the critical properties (Figure 6, Figure 7 and Figure 8) at these two stations are completely different. Moreover, the occurrence of criticality at the Kumamoto station is observed to be unique among the five stations. The time series at the Kumamoto station always reached a critical state earlier than those at the other four stations. In addition, no criticality was observed after 6 April except the second time criticality of the displacement deformation. We should explain the reason why the results at the Kumamoto station are much different from other stations. Since the time series we analyzed, in the present study, are crustal information, it implies that we have to concern the tectonic structures, here, to answer this question. We found that Kumamoto was the only station that was located at the hanging (northwest) side of Fault #2, as shown in Figure 1b. The authors of [65] used seismic data and estimated the stress field around the Kumamoto region, and also investigated the spatio-temporal variation of stress orientation. They found that the stress orientations were different across the Futagawa-Hinagu fault zone. The minimum compressive stress (σ3) axes are oriented in an approximately N–S direction on the northern side (i.e., hanging side of Fault #2 in Figure 1b), but heading NW–SE direction on the southern side. The stress axes rotate and change their orientation twice after the first foreshock and the main shock sequentially, which indicates the significant variations of crustal stress. In addition, the main shock of the Kumamoto EQ was first ruptured along Fault #1, and then the second rupture along Fault #2 was triggered a few seconds after the rupture of Fault #1 [37]. The migration of foreshocks before the Kumamoto EQ was reported by [66], which implies the stress transfers from the foreshock fault (Fault #1) to the main shock faults (both Faults #1 and #2) during the foreshock sequence. Since the tectonic structures and stress distribution are quite complicated around the two ruptured faults, we believe the two stations of Jounan and Kumamoto have some differences in approaching criticality.

In addition, the kinematic rupture process and coseismic displacement of the main shock and foreshocks were resolved in [67] on the basis of a joint inversion using GNSS, strong motion (seismometer), synthetic aperture radar, and surface offset data. The paper [67] reported an ~10 km long gap of aftershock activities around the northeast end of Fault #2, due to the increase of coseismic coulomb stress. This area of low seismic activity had weak material properties of high temperature, low resistivity, low density, and low shear wave velocity. These properties further suggested the presence of partial melting near the Aso volcano. Beyond the gap, the aftershock activities extended further northeastward, toward the middle of Oita Prefecture, and an M5.7 (estimate value) aftershock occurred right after the main shock [68]. The area around the Aso volcano was not only the end of the main shock rupture but also a singular place for the aftershock activity. The Aso GNSS station that we used, in the present study, was located exactly in the aseismic area. From Figure 6, we found that the GNSS deformation approached a criticality at every station in early April. However, critical states were present at other stations, except for Aso, in the middle of April. In addition, as shown in Figure 8, the B2 band fluctuations at the Aso station approached a criticality in late March. The early criticality at the Aso station could be regarded as an outlier that the critical state was approached much earlier than those in middle April. Thus, an 11-day gap of criticality was found at the Aso station but not at other stations. In contrast, there was no significant discrepancy in B1 band criticality between Aso and the other stations except Kumamoto (Figure 7). The GNSS deformation and B2 fluctuations at the Aso station showed some characteristic properties of criticality, which could be related to the irregular crustal condition (e.g., partial melting) around the Aso volcano [67].

**Figure 9:**
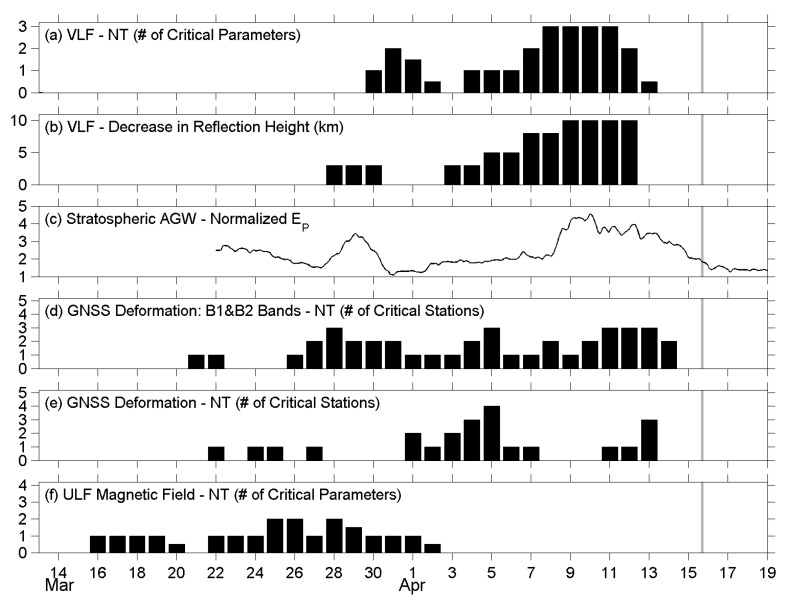
A summary of the precursory phenomena observed before the Kumamoto EQ. (**a**) The number of critical parameters for the NT analysis on the very low-frequency (VLF) data, as reported by [31]; (**b**) The decrease in VLF reflection height in the lower ionosphere. The values were adopted from [23]; (**c**) The maximum AGW potential energy (E_P_) as a proxy of stratospheric AGW activity. The values were retrieved from [24], and a normalization was performed using background mean; (**d**) A summary of critical stations for B1 and B2 fluctuations as shown in Figure 7 and Figure 8; (**e**) A summary of critical stations for deformation as shown in Figure 6; (**f**) The number of critical parameters for the NT analysis on Kanoya ULF magnetic data, after [29]. The vertical gray line in each panel indicates the occurrence of the main shock.

Next, we review the pre-seismic phenomena regarding the atmospheric oscillation channel before the Kumamoto EQ, as reported in our previous papers [23,24,29,31], and make a comparison between them and the results of the present study. Since those studies were introduced in Section 1, we have only listed some conclusions here, and Figure 9 shows the plots that contain only crucial information from each study. The lithospheric information is placed at the bottom, and the lower ionospheric information is placed at the top, as in nature. First, we look at the results of the NT analysis on the ULF magnetic field at Kanoya. Four characteristic quantities were analyzed using the NT method [29]. In Figure 9f, we simply counted the total number of critical parameters, i.e., how many parameters approached criticality, on each day during the studied period before the EQ. The criticality dates are concentrated around the second half of March, about 2–4 weeks before the EQ. Then, the results of the present study, as shown in Figure 6, Figure 7 and Figure 8, are summarized in Figure 9d,e. We counted the total number of critical stations, and the number was counted once no matter how many parameters approached criticality at the same station on that day. Figure 9e represents the number for deformation (Figure 6) and Figure 9d for B1 and B2 fluctuations, respectively. As mentioned in Section 4, on the one hand, we found the criticality of deformation appeared during the three periods of late March, early April, and just a few days before the EQ. On the other hand, the criticality of B1 and B2 fluctuations was approached continuously from 26 March to 14 April, with three peaks on 28 March, 5 April, and a few days before the EQ. In Figure 9c, the plot shows the AGW activity in the stratosphere at the EQ epicenter, as studied by [24]. The hourly E_P_ values were normalized using a long-term mean (=1) in March and April, then, the maximum value of normalized E_P_ at the stratospheric altitude of 30–50 km was taken and plotted in Figure 9c. The values in mid-March are not shown here because of the influence of meteorological effects during that period. The stratospheric AGW activity increased from the 28 to 30 March and was much more enhanced from the 8 to 15 April. Corresponding to the stratospheric AGW activity, Figure 9b is the decrease in the reflection height of VLF waves in the lower ionosphere, as computed by [23]. The reflection height descends 3 km at the end of March and the beginning of April, which is about two weeks before the EQ. However, the depletion of reflection height amplifies on the following days and becomes 10 km from the 9 to 12 April when the lower ionospheric perturbation is most violent. With the same VLF data, NT analysis was applied on three VLF quantities [31], and it was concluded that the lower ionosphere exhibits critical characteristics from two weeks before the EQ. Especially from the 8 to 11 April, all the three quantities approached criticality. Their results are also collated in Figure 9a.

Although the results mentioned in the former paragraph seem complicated, we found a general pattern from Figure 9 that the lithospheric precursor as seen in the ULF magnetic field data appears the earliest, followed by the critical states of surface deformations and correlated B1/B2 fluctuations, then, the abnormal AGW activity in the stratosphere, and lastly, the lower ionospheric perturbations as detected by both the convectional wave-hop computation and the NT analysis on the VLF data. The most important point of Figure 9 could be that the temporal evolution of the stratospheric gravity wave activity (Figure 9c) shows good similarity to that of GNSS deformation (B1 and B2 bands) (Figure 9d). The precursors were observed from the lithosphere to the lower ionosphere as time went by. In other words, the propagation of precursory phenomena can be illustrated by an arrow heading from the bottom left to the top right in Figure 9.

Before ending the discussion, we return to the goal of this paper as mentioned in Section 1. This paper aims to find the occurrence of criticality in the lithosphere, specifically, the GNSS deformation data. We have surely found the criticalities of the surface deformation, as well as the spectral components in the two frequency bands of 20–100 and 100–300 min, which are in the frequency range of AGWs. A comparison between some precursory phenomena and the results of the present study, as shown in Figure 9, reveals that the pre-seismic effects first originated in the lithosphere, and then triggered oscillations around the ground surface as a good exciter of AGWs. Furthermore, the precursory AGW activity and depletion in VLF reflection height are observed in the stratosphere and lower ionosphere, respectively, while the stress accumulated in the crust is ready to generate the EQ. Our goal for this paper was reached, and the results lend us good support to the atmospheric oscillation channel of the LAIC process.

## 6. Summary and Conclusions

As is described in detail in Section 1, our greatest concern in the field of seismo-electromagnetics is the elucidation of the LAIC process. There have been a few hypotheses proposed for the mechanism of LAIC, but the most plausible one is considered to be the AGW channel (also referred to as the atmospheric oscillation channel) [1,2].

However, unfortunately, there has been no direct evidence in the atmosphere on this AGW channel for the LAIC effect, although there has been a lot of indirect evidence accumulated on this channel [11]. We recently presented the presence of precursory AGW activity in the neutral atmosphere on the basis of a data analysis of the global ERA5 reanalysis dataset, which is considered to be a pre-seismic enhancement of AGW activity in the atmosphere as the first convincing evidence on the AGW hypothesis [24]. Similar results have also been observed for the disastrous 2011 Tohoku EQ [34].

Then, the next important point we have to study is how those pre-seismic AGWs are excited at or near the Earth’s surface, that is, the mechanism of excitation of those AGWs. Several candidates can be suggested for the exciters of AGW, including surface deformation, air temperature, air pressure, and some other parameters on the Earth’s surface. The simplest one among them is surface deformation, because GPS or GNSS position data provided us with the precursory surface deformation actually before the 2011 Tohoku EQ [32]. Furthermore, a recent work by [35] indicated that the ground vibrations during the period of 3.5–5.5 h (in the frequency range of AGWs) were very much enhanced prior to several major EQs, which is regarded as strong support to our AGW hypothesis of the LAIC process.

The purpose of this paper is to pay particular attention to the surface deformation as the first possible candidate as an exciter of AGW and to investigate whether there could have existed any criticality in the surface deformation and fluctuations in the frequency range of AGWs (AGW fluctuations) during a period from 13 March to 15 April 2016 before the 2016 Kumamoto EQs. As related to AGW fluctuations, two frequency bands of 20–100 min (B1 band) and 100–300 min (B2 band) are used, which are subjected to the NT analysis. The following is a summary of the NT results (Figure 6, Figure 7 and Figure 8):

(i) In late March, three stations, i.e., Kumamoto, Aso, and Sagara, exhibited their respective criticalities. The most critical station was Kumamoto during this period, where the deformation on displacement, as well as the two AGW bands fluctuations on height and displacement, approached criticalities.
(ii) In early April, criticalities appeared at all five stations, but the criticalities for the two AGW bands were mainly observed at the Sagara and Amakusa stations. Additionally, critical states were more often seen on latitude and longitude.
(iii) On 11–14 April or 1–4 days before the EQ main shock, criticalities were frequently observed at all five stations. The increase in the occurrence of criticality further implied the coming of the EQ.
(iv) The critical property at the Kumamoto station was different from the remaining four stations. The station is located on a different side of the ruptured fault than other stations, which could be a plausible explanation to answer this dissimilarity.

Now we compare these NT analysis results with the former corresponding analysis results for this particular Kumamoto EQs and, especially, we pay attention to the stratospheric AGW activity [24] and the lower ionospheric perturbations [31]. According to [24], the AGW potential energy in the stratosphere showed an evident increase, i.e., the AGW activity was significantly enhanced around the EQ epicenter during the week before the EQ. The authors of [24] also studied the spatial expansion of the AGW activity. Both the temporal evolution and the spatial variation of the results in [24] were consistent with the corresponding behavior of the lower ionospheric perturbations by [33]. The criticalities for the surface deformation data, as summarized in Figure 9d,e, peaked on 28 March, 5 April, and 11–13 April just before the EQ, which is likely to be consistent in time with the stratospheric result by [24]. This temporal overlapping provides us with further strong support for our AGW hypothesis, and we can consider that the surface deformation could be one possible agent of AGW exciter.

In this paper, we investigated the GNSS surface deformation data, and we found that the surface deformation could be qualitatively one candidate of AGW exciter. However, we need to perform the quantitative estimation of the effect of surface deformation on the lower ionosphere in order to explain the observed ionospheric perturbation through AGW theoretical modeling or simulations. In addition, we need to try to search for any other possible agents, and include ground parameters (air pressure, air temperature, surface latent heat flux, etc.) and near-ground parameters (such as outgoing longwave radiation) to excite the pre-seismic AGWs in the stratosphere.
